# Could eggshell membrane be an adjuvant for recombinant Hepatitis B vaccine?: A preliminary investigation

**DOI:** 10.1186/s43094-023-00481-5

**Published:** 2023-04-05

**Authors:** Parker Elijah Joshua, Charity Chinyere Ilo, Uzochukwu Gospel Ukachukwu, Damian Chukwu Odimegwu, Rita Onyekachukwu Asomadu, Timothy Prince Chidike Ezeorba

**Affiliations:** 1 Department of Biochemistry, Faculty of Biological Sciences, University of Nigeria, Nsukka, 410001 Enugu State Nigeria; 2 Department of Pharmaceutical Microbiology and Biotechnology, University of Nigeria, Nsukka, 410001 Enugu State Nigeria; 3 Department of Genetics and Biotechnology, University of Nigeria, Nsukka, 410001 Enugu State Nigeria; 4 Department of Molecular Biotechnology, School of Biosciences, University of Birmingham, Edgbaston, B15 2TT UK

**Keywords:** Adjuvant, Eggshell membrane (ESM), Immunoinformatic analysis, Immune responses, Toxicity

## Abstract

**Background:**

Despite the invasiveness of the Hepatitis B infection, its vaccines are only formulated with FDA-approved alum-based adjuvants, which poorly elicit a lasting immune response, hence the need for a more effective adjuvant system. This study evaluated the immunogenicity and toxicity of eggshell membranes (ESM) when administered as an adjuvant for the recombinant HBV vaccine (rHBsAg) in albino mice. Differential white blood cell analysis, as well as the titer measurement of Immunoglobulin G, subclass G1 and G2a on indirect ELISA, was performed to measure the immune-modulatory potentials of ESM. Moreover, analysis of the liver marker enzyme (AST and ALT) and body/liver weights was performed to ascertain the toxicity level of ESM. Finally, Immuno-informatic analysis was used to investigate the immune-modulatory potential of individual member proteins of ESM.

**Results:**

Our results showed a significant improvement in the experimental group's lymphocyte count after boost-dose administration compared to the controls, whereas there was no significant change in the granulocyte population. Furthermore, the formulations (ESM-rHBsAg) significantly improved IgG and IgG1 titers after each successive immunization. Body/liver weight and liver function showed ESM non-toxic to mice. The immunoinformatic analysis discovered ovalbumin, lysozyme-C, and UFM-1 as the member proteins of ESM with immune-modulatory activities of activating antigen-presenting cells (APC).

**Conclusion:**

This study has provided a clue into the potential valorization of eggshell membranes and their peptides as an adjuvant for vaccines such as HBV. We recommend more in-depth molecular analysis to support our findings as well as foster real-life application.

**Supplementary Information:**

The online version contains supplementary material available at 10.1186/s43094-023-00481-5.

## Background

Hepatitis B is a prevalent infectious disease caused by the DNA virus, Hepatitis B virus (HBV), which belongs to the Hepadnaviridae family [1]. Hepatitis B is widespread and has been implicated in several health complications. Globally, about 2 billion people have a serological history or are presently down with HBV infection, and 350 million are chronically infected and prone to HBV-associated liver disease [2]. Due to the hypervirulence of HBV and its high infectivity rate, the HBV vaccine was developed as a pertinent need to manage the infection [3].

Vaccination is a preparatory strategy to defend the body against the onset of HBV infections. It has been widely accepted as the most effective measure and approach to decrease the prevalence of HBV infection globally [4]. Vaccines are usually common features (antigenic pattern) among several variants of a particular infectious agent that primes the body's immune system for a prompt response against the onset of such infection. Some vaccines perform poorly and may require an immune system stimulator (adjuvants) [5].

For decades the US Food and Drug Administration (FDA) has approved the use of aluminum-containing adjuvants (alums) for primary human vaccination due to their excellent track record of safety, low cost, and application with a variety of antigens. However, alums have some limitations, especially in the vaccination with recombinant protein and small peptides [6]. Alums have been implicated in recent studies with some local reactions, zero effectiveness to some recombinant antigenic vaccines, and poor stimulatory effects to some cell-mediated immune responses, such as cytotoxic T-cell responses [7]. Achieving satisfactory immunological defense against HBV infection recently requires multiple immunization programs (MIP) with recombinant HBV surface antigen (rHBsAg) adjuvanted with Alums [8]. Generally, the vaccines shot are administered in prime and boost doses within 6 months [9]. Though, with the MIP alternatives, some success in combating HBV infection has been achieved. However, achieving and maintaining a comprehensive global vaccination of all individuals is still tricky with multiple immunizations. Hence, the need for more concerted efforts to search for more sustainable adjuvant alternatives to possibly help boost the immune response even with one shot of the vaccine. Sivakumar et al*.* [10] posited that some novel adjuvants such as MF59, which is an oil in water emulsion, ASO4, and some ISS, although reported to elicit a more robust immune response than alum-based adjuvants, have been implicated with some disadvantages such as reactogenicity and cost ineffectiveness [10].

Natural products from plant and animal origin could be another viable source of novel adjuvants with improved immune-stimulatory activities [7, 11–13]. We have reported in our previous studies the effect of snail mucin [4], Moringa Oleifera [14], chitin [5], and others as immunomodulatory agents [15–19]. In this study, we aim to investigate the immunological activities of the eggshell membrane as a potential adjuvant for HBV.

The eggshell membrane (ESM) is a film-like lining underneath the eggshell comprising several proteins, with a few minerals and fat. ESMs are often peeled off together with the shell and disposed of as waste [20]. Studies have shown that this otherwise waste product obtained from eggs, which are affordable and natural, has immunomodulatory properties with minimal side effects [21]. This present study is steered toward evaluating the immunogenicity of recombinant Hepatitis B vaccine when combined with the eggshell membrane (ESM) as a possible adjuvant for possible therapeutic use in the prevention of Hepatitis B infection. Finally, using immunoinformatic analysis, constituent member proteins of ESM were examined for the presence of immuno-modulatory peptides with the inherent ability to activate antigen-presenting cells (APC).

## Methods

### Reagents

All chemicals and reagents used for the study were of analytical grade. They included 2 M H_2_SO_4_ (JHD), 96% acetone (JHD), Alanine aminotransferase (ALT) Kit (DiaLab, Austria), aspartate aminotransferase (AST) Kit (DiaLab, Austria), carbonate-bicarbonate buffer (pH 9.5), citrate phosphate buffer (pH 5.0), DMSO (JHD, China), methanol, fat-free milk powder (Dano-Slim, Nigeria), hydrogen peroxide (JHD, China), phosphate-buffered saline (pH 7.4), Leishman stain, tetramethylbenzidine (TMB) Tablets—(SIGMA, USA), Tween 20 (Germany).

### Vaccine and secondary antibodies

Both vaccine and secondary antibodies were of analytical grade and were kept at 4 °C. The vaccine candidate used for this study is Euvax-B (LG Chemical, South Korea), a recombinant Hepatitis B surface antigen (rHBsAg) vaccine. In contrast, goat anti-mouse horseradish peroxidase-conjugated IgG, IgG1, and IgG2a immunoglobulins (Southern Biotechnology, USA) were used as secondary antibodies for iELISA.

### Experimental animal procurement

The Institutional Committee on Ethical Clearance for Animal Research gave ethical approval for the study. A total of thirty (30) female albino mice (*Mus musculus)* of about 6–8 weeks’ old were purchased from Diamond Research Farm in Nsukka, Enugu state, Nigeria. The animals were constantly fed with Guinea Commercial Feed, which had a protein content of about 30%. The animals were acclimatized for 7 days in the University Animal House. Strict adherence to the UK Animals, Scientific Procedures, Act 1986 and the National Institute of Health Guide for Care and Use of Laboratory Animals (NIH, 2011) were observed for all animal experiments in this study.

### Extraction of eggshell membrane

This was done using the method of Makkar et al*.* [22]. Specifically, the albumin and yoke of fresh unfertilized chicken eggs were siphoned out. The shell membrane attached to the eggshell was washed by mild scrubbing under running water and peeled off from the shell. The egg membranes were pulled together and washed with excess water by stirring for 2 to 3 h. Afterward, the ESM was desiccated and finally pulverized using a blender. The pulverized ESM powder was then sieved using a muslin cloth.

### Experimental design

All the animals in each experimental group were either vaccinated intramuscularly in a prime and boosted order with 2 µg of vaccine alone or in combination with the pulverized ESM (300 µg) or the pulverized ESM alone. The mice were partitioned into 5 groups, with each group having 5 mice, and the protocol is shown in Table 1.

**Table 1:** Experimental designs showing the several formulations administered to the various experimental groups

Experimental group	Formulations	Days administered
Group 1 (3DHBV)	2 µg of rHBsAg (3 doses)	1, 15 and 22
Group 2 (2DHBV)	2 µg of rHBsAg (2 doses)	1, 22
Group 3 (2DHBV + ESM)	2 µg rHBsAg + 300 µg ESM (2 doses)	1 and 22
Group 4 (3DESM)	300 µg ESM (2 doses)	1 and 22
Group 5 (3DControl)	Normal saline	1, 15 and 22

### Sera collection from experimental animals

The blood samples were collected from the mice through the retro-orbital sinus after being anesthetized mildly with death ether, as described by Parasuraman et al*.* [23], and were centrifuged at 5000 rpm for 10 min. The sera were collected with care via a micropipette, transferred into sterile Eppendorf tubes, and frozen until further biochemical analysis. The blood samples for IgG1, IgG2a, and IgG assay were collected on days 0, 14, 21, and 28.

### Antibody titre measurement

HBsAg-specific serum antibody titers (IgG1, IgG, IgG2a) were estimated by adopting an indirect enzyme-linked immunosorbent assay (iELISA) on sera obtained from each experimental animal as described by Nejati et al*.* [24]. Briefly, 96-well ELISA plates (BRANDplates®, Germany) were coated with 100 µL of rHBsAg (from vaccine) in carbonate-bicarbonate buffer pH 9.5 (coating buffer), followed by overnight incubation at 4 °C. The wells were washed 3 times with phosphate-buffered saline (PBS) pH 7.4 containing 0.05% Tween 20 (washing buffer) and blocked for 1 h at 37 °C with 5% skimmed milk in PBS (blocking buffer). Also 1:500 dilutions of sera were prepared in antibody buffer (2% skimmed milk in PBS-Tween). After washing the wells, 100 µL of each dilution was added to each well in triplicate and incubated at 37 °C for 1 h 30 min. Then, the wells were washed 3 times with washing buffer, and 100 µL of 1:1000 dilution of goat anti-mouse horseradish peroxidase (HRP)-conjugated IgG (Southern Biotechnology, USA) in antibody buffer was added as a secondary antibody. The plates were incubated for 1 h 30 min at 37 °C. Later, the wells were washed 3 times with washing buffer, and 100 µL of substrate solution (tetramethylbenzidine [TMB]-peroxide solution in citrate phosphate buffer, pH 5.0) was added incubated in a dark place at room temperature for 30 min. The reaction was stopped using 100 µL of 2 M H_2_SO_4_ (stop solution). Then, the absorbance of each well was measured at 450 nm using an automatic ELISA plate reader (GMI, USA). To detect specific IgG1 and IgG2a subclasses, goat anti-mouse IgG1 and IgG2a secondary antibodies (Southern Biotechnology, USA) were used.

### White blood cells (WBC) differential count

To determine the differential WBC count, a drop of collected blood samples was thinly smeared over a glass slide, then air-dried and stained with rapid stain kit reagents. Using a light microscope at 100X magnification, two hundred cells were counted and then classified.

### Assay of liver marker enzymes

The sera collected after day 28 were used to estimate the liver marker enzymes. The serum alanine aminotransferase (ALT) and serum aspartate aminotransferase (AST) concentrations were determined using (DiaLab, Wiener Neudorf, Austria) diagnostic kit as described by Thomas [25].

### Liver and body weight measurement

The mice were weighed every day using an electronic weighing scale. Also, on the 28th day, the liver weights were obtained after all experimental animals were killed and their livers harvested and weighed.

### Statistical analysis

Data were analyzed using one-way and two-way ANOVA on Statistical Product and Service Solutions (SPSS) software application version 18. Statistical analysis results were expressed as mean ± standard deviation. A confidence interval of 95% (p < 0.05) was adopted to ascertain significant differences between the means of the treated groups and the control.

### Retrieval of sequences for highly expressed eggshell membrane protein (HEESMP)

From several recent studies, the eggshell membrane contains more than 85% protein content [22]. The protein profile from the ESM was adopted from a study conducted by Rath et al. (Rath et al. 2017) with LC–MS/MS on Hatchery Egg Shell Membrane. The top 10 proteins were selected for this study as the highly expressed ESM protein (HEESMP). The amino acid sequence for each protein was retrieved from the NCBI protein database with their respective NCBI accession numbers (https://www.ncbi.nlm.nih.gov/).

### In silico analysis of HEESMP for immuno-modulatory peptide and antigen presenting cell epitopes (A-cell epitopes)

Adjuvants are known to elicit immune-modulatory activities by activating antigen-presenting cells. VaxinPad (https://webs.iiitd.edu.in/raghava/vaxinpad/index.php), a web server for designing peptide vaccine adjuvants, was adopted for the in silico prediction of A-cell epitopes on the surfaces of each HEESMP. The simulation was performed with a 25-mer long peptide at an SVM threshold of 0.8. Other properties of the A-Cell epitopes, such as Molecular Weight, pI Hydrophobicity, and Hydropathicity, were evaluated using Protparam tools-Expasy (https://web.expasy.org/protparam/).

### Homology modeling of HEESMP and visualization of A-cell epitope highlighted on HEESMP

The structure of HEESMP possessing A-Cell Epitope was downloaded from the Protein Data Bank from the (PDB—https://www.rcsb.org/) for those already deposited in the PDB. Moreover, structures not on the PDB were modeled with the Swiss model (https://swissmodel.expasy.org/interactive). The quality of the structure was accessed on Molprobity (http://molprobity.biochem.duke.edu/), and refinement was performed with 3Drefine (http://sysbio.rnet.missouri.edu/3Drefine/). PyMOL software (https://pymol.org/2/) was used for structural visualization and highlighting immunomodulatory peptides. For further studies, we recommend an in silico design of peptide adjuvants from immunomodulatory eggshell membrane proteins, tested on a Cimm-Simm immune simulation tool (https://150.146.2.1/C-IMMSIM/) to further validate the potential of the eggshell membrane as an adjuvant.

## Results

### Recombinant HBV vaccine adjuvanted with ESM did not significantly affect the granulocyte count of experimental animals

**Fig. 1:**
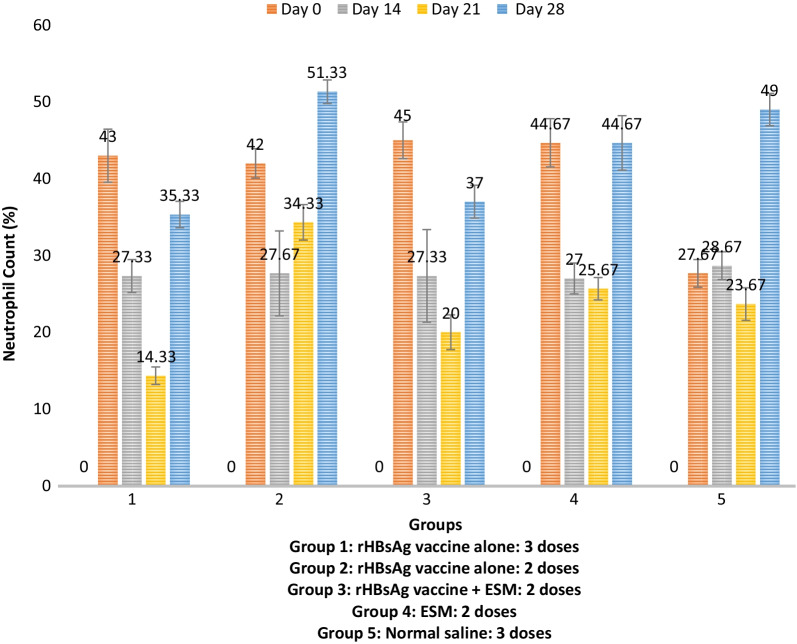
Neutrophil count from the differential WBC analysis across the experimental groups

The study conducted differential WBC analysis to estimate the proportion of Granulocytes (Neutrophils, Eosinophils, Monocytes, and Basophils) in the blood sample collected from experimental animals. Results from the analysis showed no significant changes in the levels of Eosinophils, Monocytes, and Basophils across all the experimental groups after treatment with the various formulations (Additional file 1: Tables S3, S4, and S5). However, there was a significant increase in the level of Neutrophil just after the prime (day 0) and the boost immunization (day 28), especially in the group administered with rHBsAg adjuvanted with ESM (Fig. 1 and Additional file 1: Table S1).

### Eggshell membrane as an adjuvant on rHBsAg improved the lymphocyte counts in experimental mice

**Fig. 2:**
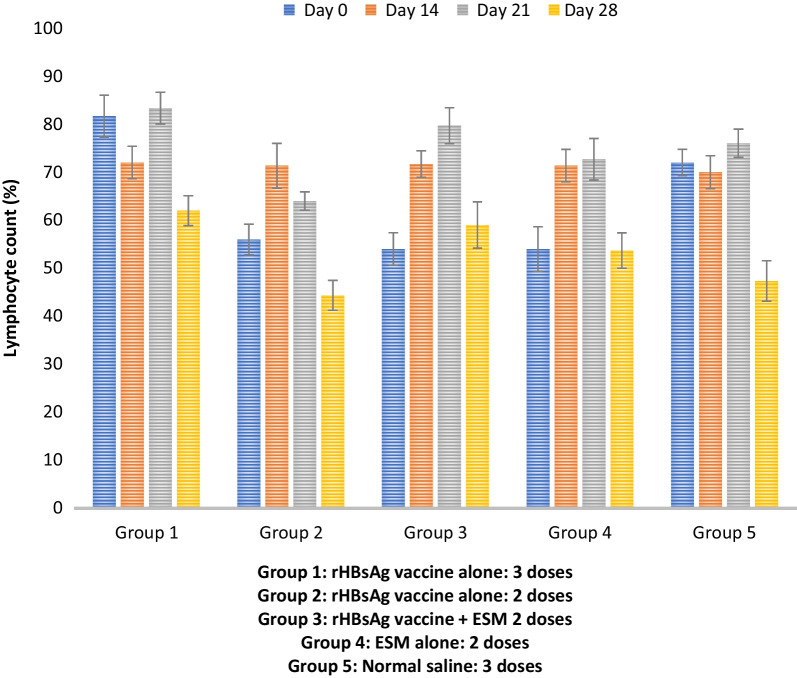
Percentage lymphocyte population from differential WBC analysis across experimental groups

The percentage lymphocyte count was estimated against other classes of WBC from the differential WBC analysis. Results in Fig. 2 showed a moderate improvement in the percentage lymphocyte population in the experimental group 3, immunized with 2 doses of rHBsAg adjuvanted with ESM (Additional file 1: Table S2). Summarily, on day 21, there was a significant increase in the percentage lymphocyte of group 3 when compared with the positive control (groups 1 and 2).

### Eggshell membrane (ESM) adjuvanted rHBsAg by showing an improvement in the group's IgG and IgG1 titer of experimental animals

**Fig. 3:**
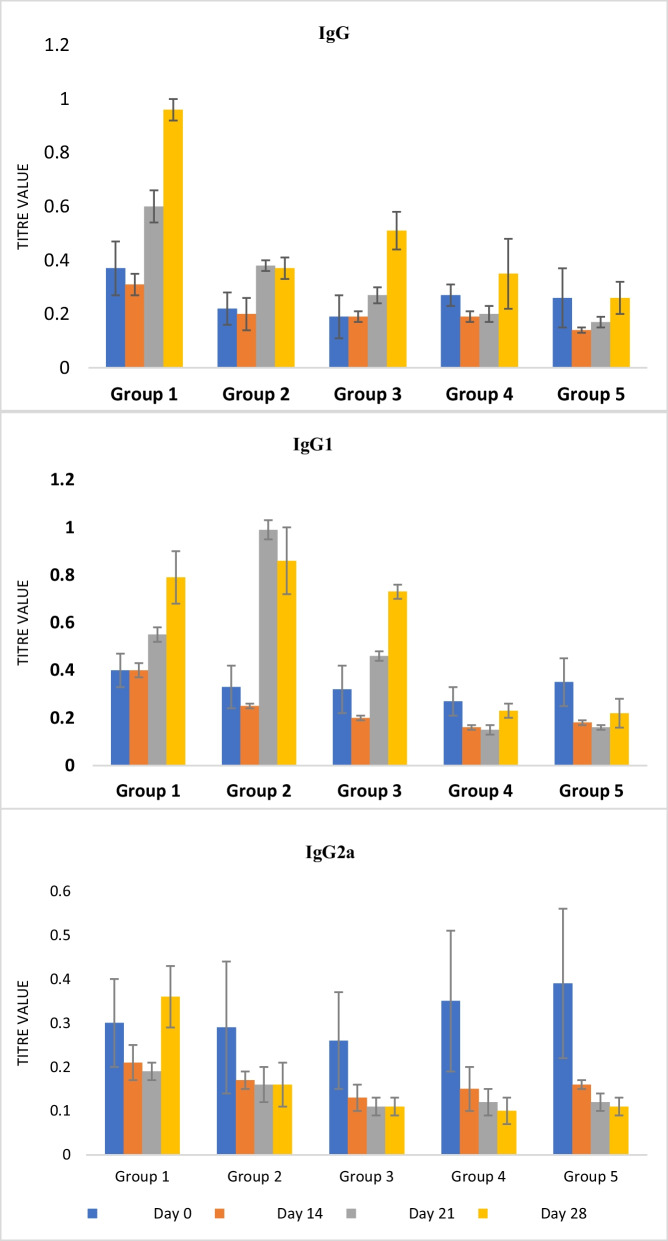
Compound graph showing the IgG, IgG1 and IgG2a titer values from iELISA experiment

The average titer value of Immunoglobulin G (IgG), Immunoglobulin G Subclass 1 (IgG1), and Immunoglobulin G Subclass 2a (IgG2a) of blood collected from experimental animals in the various groups was estimated by indirect ELISA technique. Results in Fig. 3 showed that experimental group 3 administered only 2 doses of ESM, and the vaccine expressed significantly higher IgG and IgG1 when compared to the other groups, especially the boost dose of the vaccine formulation. However, the negative controls' titer of IgG and IgG1 (groups 4 and 5) was insignificant throughout the experiment (Additional file 1: Tables S6 and S7).

Contrarily, the results in Fig. 3 showed a non-significant effect on the Immunoglobulin G Subclass 2a (IgG2a) even in the experimental group 3 and the positive control (Additional file 1: Table S8).

### Eggshell membrane (ESM) administered as an adjuvant with rHBsAg did not adversely affect the experimental animals

The liver marker enzymes were analyzed after day 28 to ascertain any toxicity from administering eggshell membranes to the animals as an adjuvant for the HBV vaccine. From the result shown in Table 1, there were no significant (*p* > 0.05) changes in the amount of AST and ALT across the group. Hence, ESM did constitute any form of toxicity to the experimental animal.

**Fig. 4:**
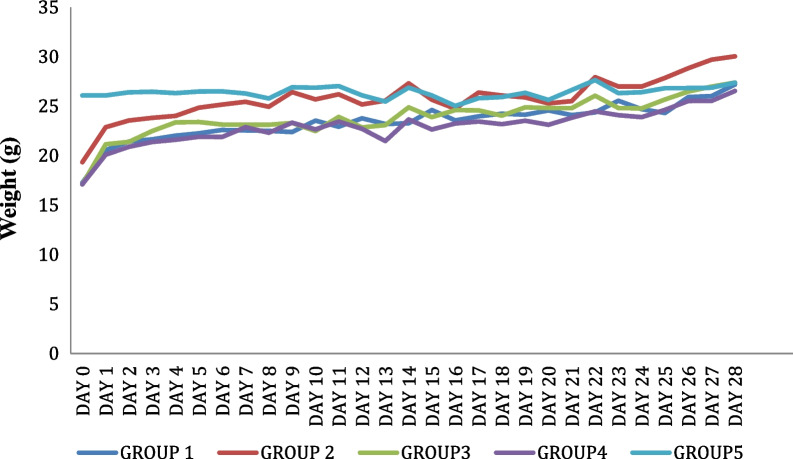
The effect of Hepatitis B vaccine modulated with eggshell membrane on the body weight of the animals

Furthermore, the result of liver weight of the animals measured in gram (g) (Table 2) and the body weight of the animals (Fig. 4) did not differ significantly, and all livers from the experimental animal had similar histology, judging from their physical liver shape and reddish-brown color.

**Table 2:** Effect of Hepatitis B vaccine modulated with eggshell membrane on liver marker enzymes and liver weight

Treatment groups	Liver marker enzymes		
	AST (U/L)	ALT (U/L)	Liver weight (g)
1	138.52 ± 7.99^a^	48.08 ± 2.79^b^	1.40 ± 0.18^ab^
2	136.51 ± 6.01^a^	41.86 ± 1.86^b^	1.67 ± 0.28^b^
3	147.16 ± 3.29^a^	46.67 ± 3.57^b^	1.31 ± 0.17^ab^
4	142.25 ± 14.80^a^	43.78 ± 4.57^b^	1.10 ± 0.16^a^
5	143.77 ± 19.52^a^	32.61 ± 4.44^a^	1.13 ± 0.19^a^

The results are expressed in means ± SD (*n* = 5). Mean values having different letters as superscripts (a, ab, b) down the group (columns) are considered to be significantly different at *p* < 0.05

### Some constituent protein of ESM had immune-modulatory (IM) epitope for activating antigen presenting cells

The constituent protein in the eggshell membrane was adopted from studies conducted by Rath et al. (2017). The study selected the top 10 proteins based on the LS-MS/MS analysis score as HEESMP. The amino acid sequence of each HEESMP was analyzed for A-cell epitopes on VaxinPad. The analysis gave only 3 out of 10 proteins to contain immuno-modulatory peptides: ovalbumin, Lysozyme C, and ubiquitin-fold modifier 1 (UFM-1) (Table 3). Ovalbumin is the most abundant protein in the spectra, with a score of 629.9. Meanwhile, Lysozyme C and UFM-1 were in the sixth and ninth positions, respectively. The immune-modulatory peptide sequence of the three proteins was also highlighted in the results in Table 3. A quick check on the properties of the peptide in Protparam Tools Expasy (https://web.expasy.org/protparam/) showed that all IM peptides are stable within the same molecular weight range (Table 3).

**Table 3:** Highly expressed eggshell membrane protein showing sequences with A-Cell epitopes and their physicochemical properties

	NCBI accession no	Protein	LC–MS/MS Scores by Rath et al. [20]	If A-cell Epitopes Present ( +) or Absent (−)	Immuno-modulatory sequence coverage	pI	Peptide MW (kDa)	GRAVY	Aliphatic index
1	QBB01285.1	Ovalbumin	629.9 (11)	+ + + +	FRADHPFLFCIKHIATNAVLFFGRCVSP	8.96	3.207	0.643	87.14
2	P01005.1	Ovomucoid	338.1 (7)	−	–	–	–	–	–
3	XP_015131596.1	Ovocleidin-116	225 (2)	−	–	–	–	–	–
4	NP_989872.1	Orosomucoid 1	167 (3)	−	–	–	–	–	–
5	XP_015151807.1	Peptidyl-prolyl cis–trans isomerase	163.5 (3)	−	–	–	–	–	–
6	NP_990612.1	Lysozyme C	129.2 (2)	+ + + +	ASVNCAKKIVSDGNGMSAWVAWRNRCKG	9.85	3.009	−0.318	59.29
7	XP_015128103.2	Keratin 8, Type II	119.1 (2)	−	–	–	–	–	–
8	NP_001012592.1	SH3 domain binding (SH3BGRL)	109.6 (2)	−	–	–	–	–	–
9	NP_001025998.1	Ubiquitin-fold modifier 1	102.1 (2)	+ + +	PAQTAGNVFLKHGSDLRIIPRDRVGSS	10.74	2892.27	0.396	86.67
10	Q6QLR1.1	Gallinacin-9	85.7 (1)	−	–	–	–	–	–

The number parenthesis is the number of peptides obtain in the LC–MS/MS analysis as conducted by Rath et al. [13]. The number of ‘ + ’ gives an indication of number of A-cell epitope in each IM protein as predicted by VaxinPad

### Homology modeling of HEESMP and visualization of A-cell epitopes highlighted in HEESMP

With the aim of investigating the position and accessibility of the A-cell epitope within the 3D structure of the Immunomodulatory HEESMP, PyMOL v2.4 was adopted to confer a distinctive appearance to the A-cell epitope. Hence, there was a need for the 3D structure of the proteins. Ovalbumin, the most abundant protein, has its structure already deposited in the PDB (with a PDB ID of 1OVA_A), while the structure of lysozyme C and UFM-1 was not on the PDB.

Homology modeling of the amino acid sequence of lysozyme C and UFM-1 on the Swiss Model gave a quality model structure used to access the various position of the A-cell epitopes on the structure. The quality assessment of both homology-modeled structures gave a Molprobity score of 1.24. Moreover, above 96% of residues in both structures were Ramachandran favored with 0% outlier (Additional file 1: Figs. S1 and S2).

**Fig. 5:**
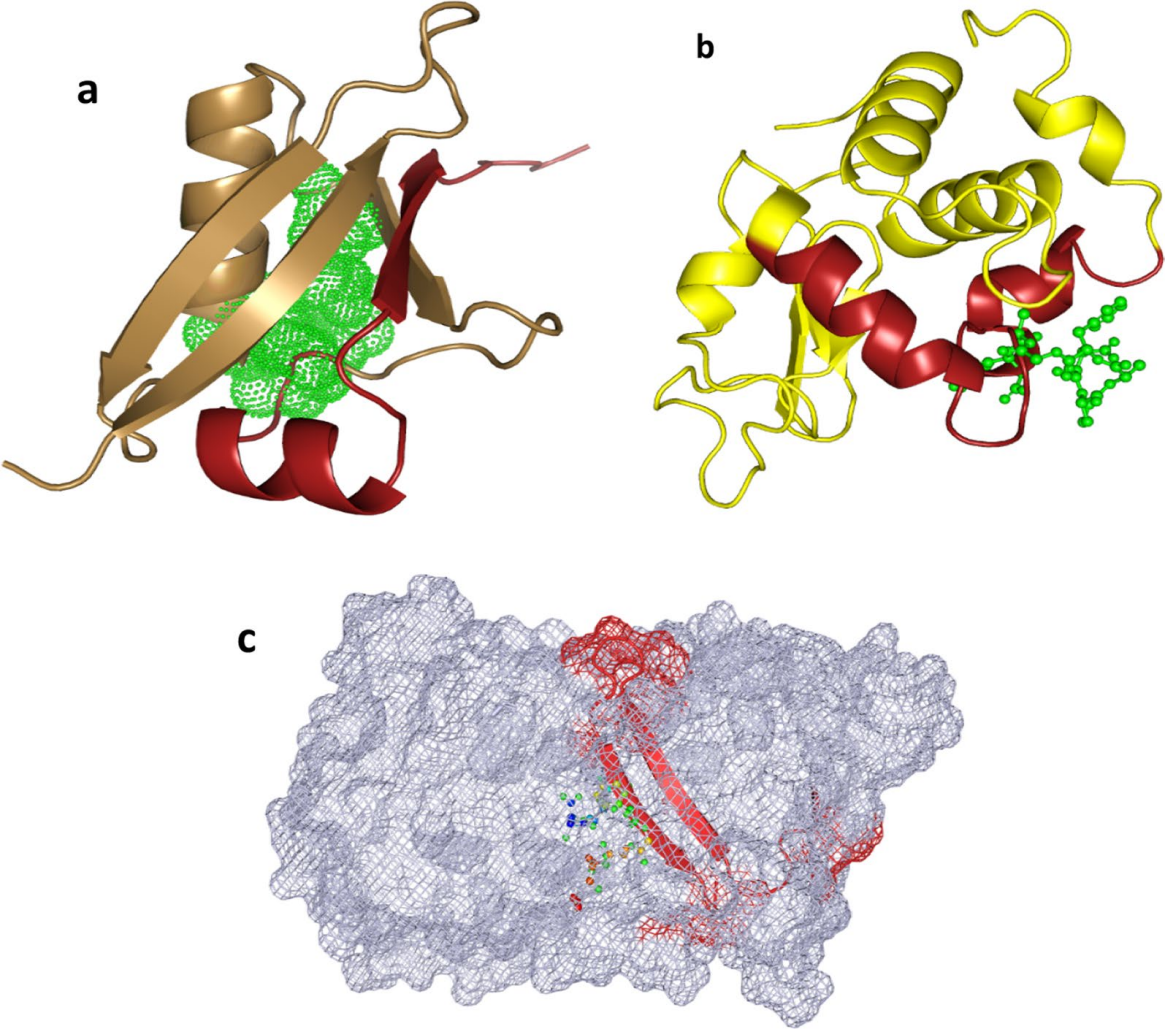
Three-dimensional structures of HEESMP (visualization using PyMOL v2.4) **a** Homology modeled structure of UFM-1, showing the A-cell epitope (IM peptide) in brick red. The interacting structure depicted in the green mesh is the vaccine (HBsAg) peptide (5YAX_c). **b)** Homology modeled structure of Lysozyme C, also showing the A-cell epitope in brick red and the green stick and ball structure is the interacting vaccine peptide (5YAX_c). **c** PDB structure of ovalbumin (1OVA_a) showing the A cell epitope in brick red embedded in the structure represented as a mesh

The structure of ovalbumin showed that the A-cell epitopes (IM peptide) are embedded in the structure's cavity. Its activities in activating APC cells may be blocked by other non-immune modulatory residues (Fig. 5c). The homolog structure of Lysozyme C and UFM-1 showed the IM peptide on the protein surface, where they can easily elicit their APC activating capacity (Fig. 5a and b).

## Discussions

Modern technologies in vaccine development must essentially assure the high safety profile of new vaccine candidates and a lower probability of causing reactogenicity in the subject in which it is administered. Vaccine development is fostered by the quest for a new vaccine that ensures a rapid and long-lasting immune response in the subject. Although advances in recombinant technology have led to the overproduction of particular and more purified antigens, lowered immunogenicity is still experienced.

Multiple immunization programs, which involve administering the vaccine for 2 – 3 doses (prime and boost doses) within a short interval, have been a significant route to addressing the lowered immunogenicity of recombinant vaccine despite their economic limitation. Alternatively, the use of adjuvants which are immunomodulatory substances is nowadays playing a crucial role. Despite the prominent role of adjuvants, only aluminum-based adjuvants have been approved by the Food and Drug Agency (FDA) of the USA and the European Medical Association (EMA), regardless of their poor actions with recombinant vaccines. Several studies and clinical trials are ongoing for improved adjuvant, especially the recombinant HBV vaccine (rHBsAg). This current study was geared toward investigating the adjuvanticity of eggshell membranes (ESM) in rHBsAg and further simulating the molecular basis of interaction between ESM proteins and rHBsAg to generate a more potent hybrid vaccine candidate possibly.

The cellular component of the immune system showed relative differences in the differential percentage count when compared across and within the various experimental groups. Neutrophils are highly phagocytic. They are the first cells to be recruited to the sites of foreign invasion [26]. For this study, the percentage of neutrophil counts was initially high and then consistently declined until day 21, after which a slight increase was observed on day 28 (Fig. 1). This is in line with the studies of Awate et al*.* [27], which opined that neutrophils are the first cells to be recruited at the site of adjuvant injection and one of the highest in numbers. This also suggests that neutrophils attract other immune cells at the injection site by producing increased amounts of chemokines and transporting antigens to the draining lymph nodes, as Bolhassani et al*.* [28]. The decline in the number of neutrophils on day 21 (Fig. 1) could be due to increased lymphocyte activity, as more antigens are presented to them by antigen-presenting cells stimulated by interaction with neutrophils [29]*.* Furthermore, the neutrophil counts of group 3 administered with 2DHBV + ESM had a non-significantly (*p* > 0.05) lower neutrophil rate than those observed in mice immunized with 3DHBV and 2DHBV.

A high lymphocyte count indicates a positive immune response as more cellular effectors are recruited [30]. The lymphocyte count was observed to increase progressively in the treatment groups by day 21, after which a decline was observed on day 28 (Fig. 2). This decline could result from clustered doses of the various vaccine formulations, as suggested by Harini et al*.* [31]. They observed that when animals that have responded maximally are given a booster dose of antigen too soon, suppression rather than enhancement of the immune response may ensue. Another plausible reason for this decline in the number of lymphocytes could be the negative effect of age on the lymphocyte count in mice. This is in accordance with the studies of Xie et al*.* [32], which explained that lymphocyte count significantly decreases with age, consequently increasing susceptibility to infectious diseases and poorer response to vaccination. The lymphocyte counts in 2DHBV + ESM immunized mice were significantly (*p* < 0.05) higher than those observed in mice immunized with a Hepatitis B vaccine 2 dose regimen. Moreover, this value was comparable to that obtained in mice immunized with Hepatitis B vaccine 3 dose regimens (Fig. 2). This suggests that 2DHBV + ESM might have elicited more lymphocyte recruitment than the conventional recombinant Hepatitis B vaccine.

For the eosinophil count, it was observed that there was a non-significant (*p* > 0.05) difference among the groups on days 21 and 28 when compared to each other (Additional file 1: Table S1). The low values observed were within the normal range of 0–7%, as described by O'Connell [33], which implies the absence of parasitic infections and hypersensitive responses [34] among the mice that were immunized with 2DHBV + ESM and also in the mice groups immunized with Hepatitis B vaccine alone. However, the progressive increase in this number could result from the recruitment of eosinophils by alums contained in the vaccine formation, which helps in transporting antigens for presentation in the lymph nodes, as suggested by Awate et al*.* [27].

The few monocyte cells counted were significantly (*p* < 0.05) higher in the 2DHBV + ESM experimental group when compared to groups 1 (3DHBV) and group 2 (2DHBV) on days 21 and 28. More so, there was a progressive increase in monocytes and the number of days (Additional file 1: Table S2). This indicates an active immune response as the antigen-presenting cells (APCs), macrophages, and dendritic cells are activated, which in turn ushers in the adaptive immune response for antibody production [35]. Similarly, basophils were absent throughout the study (Additional file 1: Table S3), indicating no allergic reaction [36] to the administration of 2DHBV + ESM or HBV vaccines in isolation. This is also in consonance with the report of O'Connell [33] that basophils were rarely present in the peripheral blood of mice.

Results from the liver marker enzymes (AST and ALT) measurement showed similar values for all the experimental groups (Table 2). Similarly, the body weights of the animals and the liver weight and histology were very similar, indicating that the ESM did not in any way result in any form of toxicity to the animals (Table 2 and Fig. 4). Similar inferences were documented by Wedekind et al*.* [37], asserting that eggshell membranes had no adverse effect on experimental mice's body and organ weights. Furthermore, the values obtained for the AST and ALT were all within the normal range for healthy mice. This indicated that there was no hepatotoxicity induced in the mice. The ALT and AST values agreed with those obtained by Ruff et al*.* [38] and Jia et al*.* [39]*,* respectively.

The adaptive humoral response is estimated by the relative titer of IgG, IgG1, and IgG2a antibodies. These antibody subclasses were chosen as better serologic markers for Hepatitis B infection and vaccination [40]. In the immunoglobulin class, IgG is very specific to viral infections and remains in the system in wait for secondary infections [41]. Figure 3 (Additional file 1: Table S6) indicates that there is no significant (*p* > 0.05) increase across all the treatment groups on day 14 (after the primer dose). This is in consonance with the works of Wang et al. [42], which showed a slow IgG production after the first immunization. Day 21 (7 days after the first booster dose indicated a significant (*p* < 0.05) increase when 2DHBV + ESM immunized mice were compared to mice immunized with the Hepatitis B vaccine. 2 doses of regimen and non-significant (*p* > 0.05) decrease when compared to the 3 doses of regimen (group 1). The results show that the HBV vaccine modulated with ESM elicited more IgG antibody production than the conventional Hepatitis B vaccine. In addition, there was a progressive significant (*p* < 0.05) increase within Groups 1, 2, and 3 across the various days. This suggests that there was a slow but progressive antigen release with the help of possible formation of depot sites by the adjuvant resulting in a steady and progressive production of antibodies. This agrees with the report of Harini et al*.* [31].

The IgG1 marker for the Th2 response is mainly elicited by alum-based adjuvants [43]. Group 3 (2DHBV + ESM) immunized mice maintained higher IgG1 titers relative to mice immunized with both 2 and 3 doses of the HBV vaccine for 28 days. This indicates that Hepatitis–B vaccine modulated with the eggshell membrane at 2 doses elicited more IgG1 antibody production than the conventional Hepatitis B vaccine adjuvanted with alum at both 2 and 3 doses; hence it potentiates a more robust Th2 response than the alum. This is in line with Benson et al*.* [44], in which eggshell membranes produce IL-4, IL-5, IL-6, and IL-10 indicators of Th2 responses. Finally, there was a progressive significant (*p* < 0.05) increase within groups 1, 2, and 3 across days 14, 21, and 28. The progressive increase in IgG antibody production could also be attributed to depot formation.

Although IgG2a was not significantly produced, there were significantly (*p* < 0.05) elevated levels in mice that were immunized with 2DHBV + ESM when compared to mice that were immunized with HBV vaccine (groups 1 and 2) from days 21 to 28. IgG2a, an indicator of Th1 immune response, is better produced from the ESM adjuvanted vaccine than the adjuvanted alum vaccine. This is as important as Th1 is needed for attacks on intracellular pathogens/antigens [45]. This observation is in accordance with the work of Benson et al*.* [44], in which eggshell membrane led to the production of IFN-γ, an indicator of Th1 responses. Finally, Group 3 recorded a significant (*p* < 0.05) increase across days 14, 21, and 28, which is the same trend of progressive increase. This could also be attributed to depot formation, as in IgG antibody production.

ESM contains several significant proteins. A study by Rath et al.[20] identified about 41 protein types in the eggshell membrane, with ovalbumin being the most abundant. Table 3 shows that only three proteins from the HEESMP—Ovalbumin, Lysozyme C, and UFM-1, had immunomodulatory peptides in their structures. Examining the position of the IM peptide on their 3D structure was necessary to suggest if the peptide will probably elicit immune-modulatory activities. While the structure of Lysozyme C and UFM-1 had its IM peptide on the surface (Fig. 5a and b), the IM peptide of ovalbumin was deeply embedded in the structure (Fig. 5c).

Several studies have shown lysozyme-C to possess immune-modulatory activities, especially for bacterial immunity. Lysozyme C is known to cause bacterial killing by degrading its peptidoglycan cell wall and using peptidoglycan debris to activate recognizable pattern receptors for future immunity [46]. Moreover, recent studies using RNA-Seq experiment have pointed out that Lysozyme C possesses not only bacterial killing activities but also performs transcriptional modulation of TNF-α/IL-1β pathway genes in U937 monocytes, which are crucial for viral immunity [47]. Hence, Lysozyme in the eggshell membrane formulation may be one of the factors for an improved immune response when administered with HBsAg in mice.

Ubiquitin fold modifier-1 (UBF-1) was a recently discovered ubiquitin-like protein with sparsely available information on its functions [48]. Generally, the UBL family of proteins covalently modifies specific proteins to their functional state. Moreover, a recent study found UBF-1 to play a crucial role in immune modulation and inflammatory responses, especially for diabetic conditions [49–51]. Since our in silico analysis gave a plausible tentative clue to UBF-1 possessing immune modulatory peptides (Fig. 5a), there is a need for future studies to investigate the role of UBF-1 in immunity, especially against viruses.

Ovalbumin, the most abundant protein in ESM, may not have contributed to the overall immune modulatory of the eggshell protein due to the embedded IM peptide, which may not be easily accessible for immune activities. Similarly, several studies have shown ovalbumin to confer immune tolerance, especially when administered via the oral route [52]. Moreover, a unique study by Plunkett et al.[53] showed that ovalbumin stimulates immune responses that are best administered by trans-dermal, intraperitoneal, and at least via oral ingestion. Another study by Basto et al.[54] reported an improved immune response with ovalbumin when fused with bacterial OprI lipoprotein. The fusion process may lead to exposure of the embedded IM peptide to ovalbumin, hence the positive immune-modulatory activities.

We recommend for future study adjuvant peptide design from IM proteins of ESM. The in silico approach may first be adopted to design an adjuvant peptide that is non-allergic, non-toxic, and highly immunogenic, especially when administered with the HBV vaccine. Recombinant technology can be used to overexpress peptides in yeast for further experimental analysis.

## Conclusion

This study has provided a clue into the potential valorization of eggshell membranes and their peptides as an adjuvant for vaccines such as HBV. We have shown that when co-administered with recombinant HBV vaccine, eggshell membrane caused a significant improvement in the lymphocyte counts, Immunoglobulin G (IgG), and Immunoglobulin G Subclass 1. Moreover, ESM did not constitute any toxicity to the experimental animals, and other immunological markers were not adversely affected. Immunoinformatic studies have identified interesting bioactive proteins and peptides with immune-modulatory function, potential avenues for future research. We recommend more in-depth molecular analysis to support our findings and foster real-life applications.

## Supplementary Information

**Additional file 1**. Supplementary file.

## Data Availability

All data reported in this work are available.

## Abbreviations

ESM: Eggshell membrane
WBC: White blood cells
HBV: Hepatitis B virus
rHBsAg: Recombinant Hepatitis B vaccine
IgG: Immunoglobulin G
IgG1: Immunoglobulin G subclass 1
IgG2a: Immunoglobulin G subclass 2a
iELISA: Indirect enzyme-linked immunosorbent assay
AST: Aspartate aminotransferase
ALT: Alanine transaminase
UFM-1: Ubiquitin-fold modifier 1
HEESMP: Highly expressed eggshell membrane protein
