# Supplementary material for: Could eggshell membrane be an adjuvant for recombinant Hepatitis B vaccine?: A preliminary investigation
Source: Futur J Pharm Sci. 2023 Apr 5;9(1):28. doi: 10.1186/s43094-023-00481-5 (PMC10074367; doi:10.1186/s43094-023-00481-5)
Supplement: Supplementary file 1 — Additional file 1. Supplementary file. [file 43094_2023_481_MOESM1_ESM.docx]

**SUPPLEMENTARY INFORMATION**

**ON**

**Could eggshell membrane be an adjuvant for recombinant Hepatitis B vaccine?: A preliminary experimental and immunoinformatic investigation**

**Supplementary Table 1** Effects of Hepatitis –B Vaccine Modulated with Egg Shell Membrane on Neutrophil Count

| \| **Treatment**  **group** \| **Day 0** \| **Day 14** \| **Day 21** \| **Day 28** \| \| --- \| --- \| --- \| --- \| --- \| \| 1 \| 43.00 ± 17.35^aB^ \| 27.33 ±10.69^aAB^ \| 14.33 ± 1.15^aA^ \| 35.33±11.72^aAB^ \| \| 2 \| 42.00 ±9.54^aAB^ \| 27.67± 13.65^aA^ \| 34.33±2.31^bAB^ \| 51.33 ± 7.64^aB^ \| \| 3 \| 45.00 ± 9.54^aB^ \| 27.33 ± 6.03^aAB^ \| 20.00 ± 11.36^abA^ \| 37.00 ± 8.54^aAB^ \| \| 4 \| 44.67 ±12.50^aA^ \| 27.00 ± 10.00^aA^ \| 25.67 ± 7.23^abA^ \| 44.67 ± 14.19^aA^ \| \| 5 \| 27.67 ± 7.23^Aa^ \| 28.67 ± 9.07^aA^ \| 23.67 ± 10.60^abA^ \| 49.00 ± 10.53^aB^ \|   **Neutrophil % across and within groups** |
| --- | --- | --- | --- | --- | --- | --- | --- | --- | --- | --- | --- | --- | --- | --- | --- | --- | --- | --- | --- | --- | --- | --- | --- | --- | --- | --- | --- | --- | --- | --- |

Results are expressed in Means ± SD (n = 3)

Mean values having different lower case letters as superscripts (a, ab, b) across the groups (from top to bottom of the column) and those having different upper case letters as superscripts (A,AB,B) across the rows are considered to be significantly different at p< 0.05.

Group 1: 2µg of hepatitis B vaccine (HBV) (3 doses)

Group 2: 2µg of hepatitis B vaccine (HBV) (2 doses)

Group 3: 2µg of (HBV) + (300µg) Egg shell membrane ESM (2 doses)

Group 4: (300µg) Egg shell membrane (ESM) alone

Group 5: Control (Normal Saline)

**Supplementary Table 2** Effects of Hepatitis –B Vaccine Modulated with Egg Shell Membrane on Lymphocyte Count

| **Lymphocytes % across and within groups** |
| --- |

| **Treatment**  **group** | **Day 0** | **Day 14** | **Day 21** | **Day 28** |
| --- | --- | --- | --- | --- |
| 1 | 56.67 ± 17.47^Aa^ | 72.00 ± 9.54^aAB^ | 83.33 ± 2.31^bB^ | 62.00 ± 10.15^aAB^ |
| 2 | 56.00 ± 8.72^aAB^ | 71.33 ± 14.57^aB^ | 64.00 ± 1.00^aB^ | 44.33 ± 7.09^aA^ |
| 3 | 54.00 ± 8.00^aA^ | 71.66 ± 7.02^aBC^ | 79.67 ± 11.01^bC^ | 59.00 ±7.81^aAB^ |
| 4 | 54.00 ± 10.53^aA^ | 71.33 ± 9.50^aA^ | 72.67 ± 6.66^abA^ | 53.67 ± 14.64^aA^ |
| 5 | 72.00 ± 6.93^aB^ | 70.00 ± 9.64^aB^ | 76.00 ± 11.13^abB^ | 47.33 ± 11.15^aA^ |

Results are expressed in Means ± SD (n = 3)

Mean values having different lower case letters as superscripts (a, ab, b) across the groups (from top to bottom of the column) and those having different upper case letters as superscripts (A,AB,B,BC,C) across the rows are considered to be significantly different at p < 0.05.

Group 1: 2µg of hepatitis B vaccine (HBV) (3 doses)

Group 2: 2µg of hepatitis B vaccine (HBV) (2 doses)

Group 3: 2µg of (HBV) + (300µg) Egg shell membrane ESM (2 doses)

Group 4: (300µg) Egg shell membrane (ESM) alone

Group 5: Control (Normal Saline)

**Supplementary Table 3** Effect of Hepatitis-B Vaccine Modulated with Egg Shell Membrane on Eosinophil Count

| **Treatment**  **Group** | **Day 0** | **Day 14** | **Day 21** | **Day 28** |
| --- | --- | --- | --- | --- |
| 1 | 0.00 ± 0.00^aA^ | 0.33 ± 0.58^abA^ | 0.33 ± 0.58^aA^ | 0.00 ± 0.00^aA^ |
| 2 | 0.33 ± 0.58^abA^ | 1.00 ± 0.00^bA^ | 1.33 ±1.15^aA^ | 0.67 ± 1.15^aA^ |
| 3 | 0.00 ± 0.00^aA^ | 0.33 ± 0.58^abA^ | 0.33 ± 0.58^abA^ | 0.00 ± 0.00^aA^ |
| 4 | 0.00 ± 0.00^aA^ | 1.33 ± 0.58^bB^ | 1.33 ± 0.58^aB^ | 0.00 ± 0.00^aA^ |
| 5 | 0.00 ± 0.00^aA^ | 0.33 ± 0.58^aA^ | 1.33 ± 0.58^bB^ | 0.67 ± 0.58^aAB^ |

Results are expressed in Means ± SD (n = 3)

Mean values having different lower case letters as superscripts (a, ab, b) across the groups (from top to bottom of the column) and those having different upper case letters as superscripts (A,AB,B) across the rows are considered to be significantly different at p < 0.05.

Group 1: 2µg of hepatitis B vaccine (HBV) (3 doses)

Group 2: 2µg of hepatitis B vaccine (HBV) (2 doses)

Group 3: 2µg of (HBV) + (300µg) Egg shell membrane ESM (2 doses)

Group 4: (300µg) Egg shell membrane (ESM) alone

Group 5: Control (Normal Saline)

**Supplementary Table 4:** Effects of Hepatitis-B Vaccine Modulated with Egg Shell Membrane on Monocyte Count

| **Monocytes % across and within groups** |
| --- |

| **Treatment**  **group** | **Day 0** | **Day 14** | **Day 21** | **Day 28** |
| --- | --- | --- | --- | --- |
| 1 | 0.00 ± 0.00^aA^ | 0.33 ± 0.58^aA^ | 0.67 ± 1.15^aA^ | 3.00 ± 2.64^aA^ |
| 2 | 0.33 ± 0.58^aA^ | 0.67 ± 0.58^aA^ | 1.00 ± 1.00^aA^ | 3.33 ± 0.58^aB^ |
| 3 | 0.67 ± 0.58^aA^ | 1.00 ± 1.00^aA^ | 2.00 ± 1.73^bAB^ | 4.00 ± 3.00^aB^ |
| 4 | 0.00 ± 0.00^aA^ | 0.33 ± 0.58^aA^ | 1.33 ± 2.31^aA^ | 1.67 ± 0.58^aA^ |
| 5 | 0.00 ± 0.00^aA^ | 0.00 ± 0.00^aA^ | 0.33 ± 0.56^aA^ | 3.00 ± 2.64^aB^ |

Results are expressed in Means ± SD (n = 3)

Mean values having different lower case letters as superscripts (a, ab, b) across the groups (from top to bottom of the column) and those having different upper case letters as superscripts (A,AB,B) across the rows are considered to be significantly different at p < 0.05.

Group 1: 2µg of hepatitis B vaccine (HBV) (3 doses)

Group 2: 2µg of hepatitis B vaccine (HBV) (2 doses)

Group 3: 2µg of (HBV) + (300µg) Egg shell membrane ESM (2 doses)

Group 4: (300µg) Egg shell membrane (ESM) alone

Group 5: Control (Normal Saline)

**Supplementary Table 5** Effects of Hepatitis –B Vaccine Modulated with Egg Shell Membrane on Basophil Count

| **Basophils % across and within groups** |
| --- |

| **Treatment**  **Group** | **Day 0** | **Day 14** | **Day 21** | **Day 28** |
| --- | --- | --- | --- | --- |
| 1 | 0.00 ± 0.00 | 0.00 ± 0.00 | 0.00 ± 0.00 | 0.00 ± 0.00 |
| 2 | 0.00 ± 0.00 | 0.00 ± 0.00 | 0.00 ± 0.00 | 0.00 ± 0.00 |
| 3 | 0.00 ± 0.00 | 0.00 ± 0.00 | 0.00 ± 0.00 | 0.00 ± 0.00 |
| 4 | 0.00 ± 0.00 | 0.00 ± 0.00 | 0.00 ± 0.00 | 0.00 ± 0.00 |
| 5 | 0.00 ± 0.00 | 0.00 ± 0.00 | 0.00 ± 0.00 | 0.00 ± 0.00 |

Results are expressed in Means ± SD (n = 3)

Group 1: 2µg of hepatitis B vaccine (HBV) (3 doses)

Group 2: 2µg of hepatitis B vaccine (HBV) (2 doses)

Group 3: 2µg of (HBV) + (300µg) Egg shell membrane ESM (2 doses)

Group 4: (300µg) Egg shell membrane (ESM) alone

Group 5: Control (Normal Saline)

**Supplementary Table 6** Effects of Hepatitis–B Vaccine Modulated with Egg Shell Membrane on IgG Titre

| **IgG titre across and within groups** | | | | |
| --- | --- | --- | --- | --- |
| **Treatment**  **Groups** | **Day 0** | **Day 14** | **Day 21** | **Day 28** |
| 1 | 0.83 ± 0.12^bA^ | 1.14 ± 0.26^Aa^ | 2.26 ± 0.09^cB^ | 2.44 ± 0.51^bB^ |
| 2 | 0.50 ± 0.08^aA^ | 0.69 ±0.27^Aa^ | 1.51 ± 0.12^abB^ | 1.70 ± 0.07^bB^ |
| 3 | 0.47± 0.03^aA^ | 0.63 ± 0.24^aA^ | 2.26 ± 0.07^cB^ | 2.43 ± 0.47^bB^ |
| 4 | 0.33 ± 0.05^aA^ | 0.44 ± 0.02^aA^ | 0.65 ± 0.48^aA^ | 1.31 ± 0.96^abA^ |
| 5 | 0.46 ± 0.10^aA^ | 0.56 ± 0.05^Aa^ | 0.72 ± 0.62^aA^ | 0.88 ± 0.65^aA^ |

Results are expressed in Means ± SD (n = 3)

Mean values having different lower case letters as superscripts (a, ab, b,c) across the groups (from top to bottom of the column) and those having different upper case letters as superscripts (A,B) across the rows are considered to be significantly different at p < 0.05.

Group 1: 2µg of hepatitis B vaccine (HBV) (3 doses)

Group 2: 2µg of hepatitis B vaccine (HBV) (2 doses)

Group 3: 2µg of (HBV) + (300µg) Egg shell membrane ESM (2 doses)

Group 4: (300µg) Egg shell membrane (ESM) alone

Group 5: Control (Normal Saline)

**Supplementary Table 7** Effects of Hepatitis –B Vaccine Modulated with Egg Shell Membrane on IgG1 Titre

| **IgG1 titre across and within groups** | | | | |
| --- | --- | --- | --- | --- |
| **Treatment**  **groups** | **Day 0** | **Day 14** | **Day 21** | **Day 28** |
| 1 | 0.74 ± 0.31^aA^ | 0.93 ± 0.08^Da^ | 2.70 ± 0.03^dB^ | 2.67 ± 0.38^bB^ |
| 2 | 0.37 ± 0.17^aA^ | 0.47 ± 0.01^cA^ | 2.20 ± 0.06^bB^ | 1.94 ± 0.04^bB^ |
| 3 | 0.41 ± 0.05^aA^ | 0.40 ± 0.04^bcA^ | 2.80 ± 0.07^dB^ | 2.76 ± 0.27^bB^ |
| 4 | 0.24 ± 0.04^aA^ | 0.40 ± 0.08^aA^ | 0.37± 0.05^bA^ | 0.64 ± 0.10^aA^ |
| 5 | 0.11 ± 0.08^aA^ | 0.10 ± 0.04^aA^ | 0.14 ± 0.03^aA^ | 0.17 ± 0.12^aA^ |

Results are expressed in Means ± SD (n = 3)

Mean values having different lower case letters as superscripts (a, b, bc, c, d) across the groups (from top to bottom of the column) and those having different upper case letters as superscripts (A,B) across the rows are considered to be significantly different at p < 0.05.

Group 1: 2µg of hepatitis B vaccine (HBV) (3 doses)

Group 2: 2µg of hepatitis B vaccine (HBV) (2 doses)

Group 3: 2µg of (HBV) + (300µg) Egg shell membrane ESM (2 doses)

Group 4: (300µg) Egg shell membrane (ESM) alone

Group 5: Control (Normal Saline)

**Supplementary Table 8:** Effects of Hepatitis B Vaccine Modulated with Egg Shell Membrane on IgG2a Titre

| **IgG2a titre across groups** | | | | |
| --- | --- | --- | --- | --- |
| **Treatment groups** | **Day 0** | **Day 14** | **Day 21** | **Day 28** |
| 1 | 0.28 ±0.06^aA^ | 0.29 ± 0.04^aA^ | 0.28 ± 0.01^aA^ | 0.28 ± 0.01^aA^ |
| 2 | 0.23 ±0.04^aA^ | 0.31 ± 0.06^aA^ | 0.28 ± 0.05^aA^ | 0.28 ± 0.05^aA^ |
| 3 | 0.25 ±0.04^aA^ | 0.34 ±0.13^aAB^ | 0.42 ± 0.05^bB^ | 0.42 ± 0.06^bB^ |
| 4 | 0.23 ±0.01^aA^ | 0.26 ± 0.05^aAB^ | 0.31 ± 0.02^aB^ | 0.31 ± 0.02^aB^ |
| 5 | 0.33 ±0.06^aA^ | 0.31 ± 0.02^aA^ | 0.35 ± 0.03^aA^ | 0.35 ± 0.03^aA^ |

Results are expressed in Means ± SD (n = 3)

Mean values having different lower case letters as superscripts (a, b) across the groups (from top to bottom of the column) and those having different upper case letters as superscripts (A,AB,B) across the rows are considered to be significantly different at p < 0.05.

Group 1: 2µg of hepatitis B vaccine (HBV) (3 doses)

Group 2: 2µg of hepatitis B vaccine (HBV) (2 doses)

Group 3: 2µg of (HBV) + (300µg) Egg shell membrane ESM (2 doses)

Group 4: (300µg) Egg shell membrane (ESM) alone

Group 5: Control (Normal Saline)


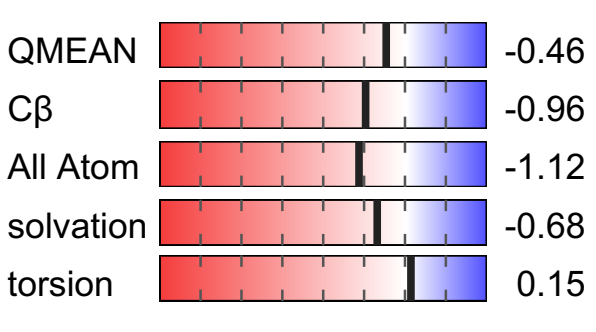


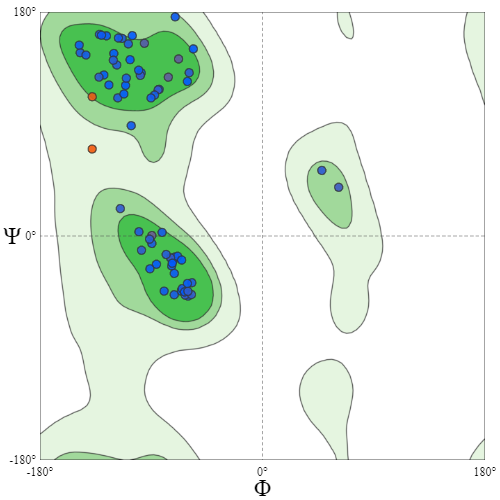

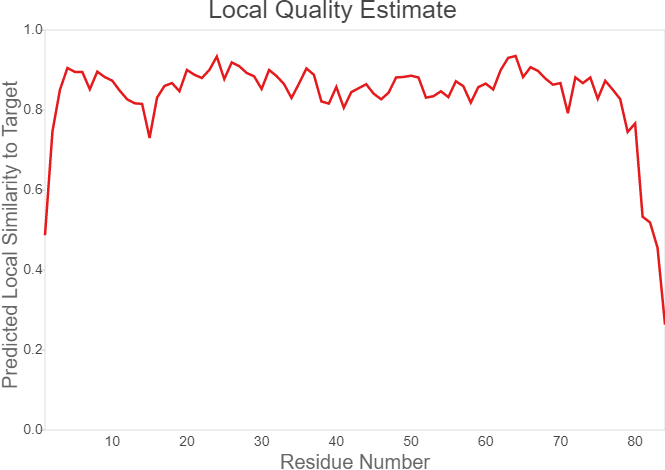

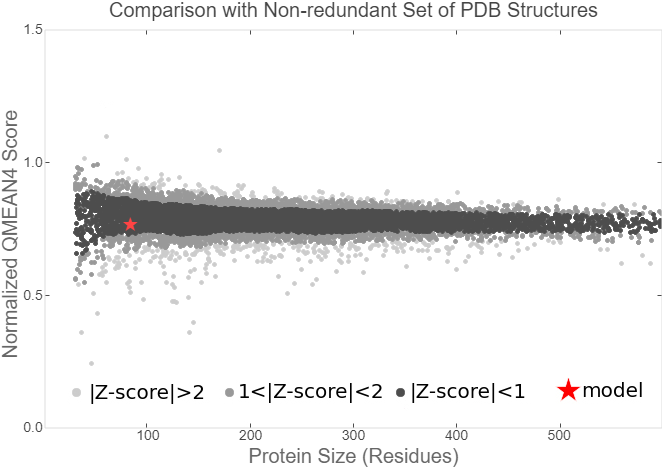


**a**

**b**

**c**

**d**

**Supplementary Figure 1**  Quality assessment of the homology modelled structure of Ubiquitin Fold Modifier-1 (Accession number - NP_001025998.1) **a)** Ramachandran plot arising from the protein secondary structure, which favoured 98.7 % of the residues with 0 % outlier. **b)** Representation of the local quality estimate of the modelled structure, which was on average above 0.8 **c)** Representation the location of structure amongst other non-redundant set of PDB structures. The red star represents the model structure which appear in a region of satisfactory Z-score. d). overall quality summary of the modelled structure. The thick black line represents the position the structure in quality tray (having the blue region as the most accurate and the red region as the least accurate).


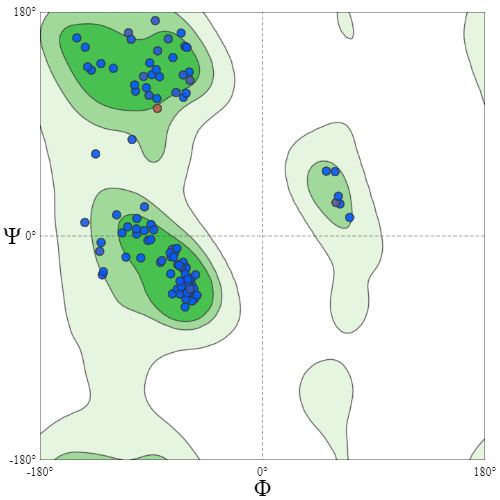

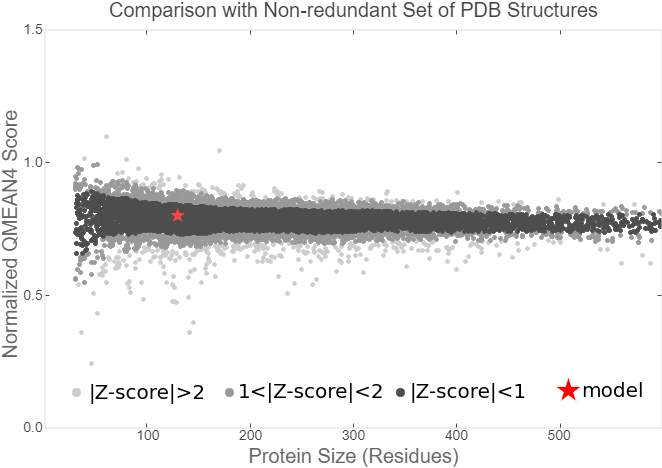

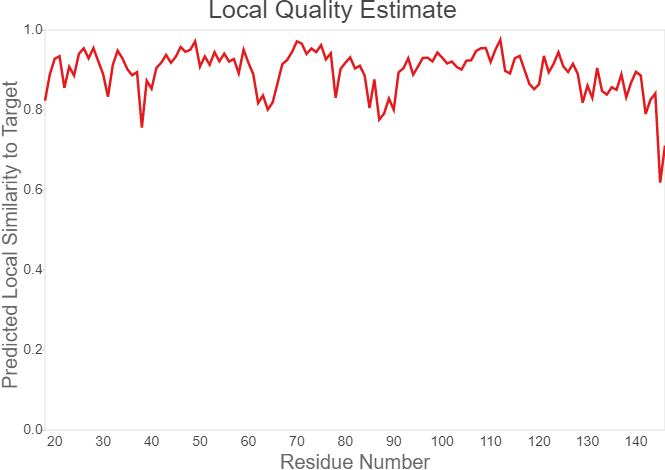

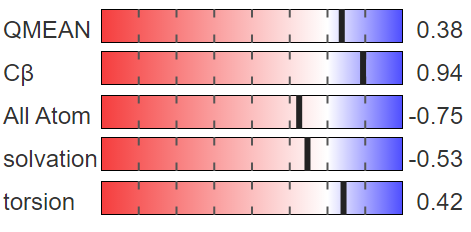


**a**

**b**

**c**

**d**

**Supplementary Figure 2:**  Quality assessment of the homology modelled structure of Lysozyme C (Accession number - NP_990612.1) **a)** Ramachandran plot arising from the protein secondary structure, which favoured 96.85% of the residues with 0% Ramachandran outlier. **b)** Representation of the local quality estimate of the modelled structure, which was on average above 0.9 **c)** Representation the location of structure amongst other non-redundant set of PDB structures. The red star represents the model structure which appear in a region of satisfactory Z-score. d). overall quality summary of the modelled structure. The thick black line represents the position the structure in quality tray (having the blue region as the most accurate and the red region as the least accurate).
